# *Trypanosoma brucei gambiense* group 2 experimental *in vivo* life cycle: from procyclic to bloodstream form[Fn FN1]

**DOI:** 10.1051/parasite/2024009

**Published:** 2024-03-22

**Authors:** Paola Juban, Jean-Mathieu Bart, Adeline Ségard, Vincent Jamonneau, Sophie Ravel

**Affiliations:** INTERTRYP, Université de Montpellier, Cirad, IRD Montpellier France

**Keywords:** *Trypanosoma brucei gambiense*, Procyclic form, Bloodstream form, *Glossina*, Life cycle

## Abstract

*Trypanosoma brucei gambiense* (*Tbg*) group 2 is a subgroup of trypanosomes able to infect humans and is found in West and Central Africa. Unlike other agents causing sleeping sickness, such as *Tbg* group 1 and *Trypanosoma brucei rhodesiense*, *Tbg*2 lacks the typical molecular markers associated with resistance to human serum. Only 36 strains of *Tbg*2 have been documented, and therefore, very limited research has been conducted despite their zoonotic nature. Some of these strains are only available in their procyclic form, which hinders human serum resistance assays and mechanistic studies. Furthermore, the understanding of *Tbg*2’s potential to infect tsetse flies and mammalian hosts is limited. In this study, 165 *Glossina palpalis gambiensis* flies were experimentally infected with procyclic *Tbg*2 parasites. It was found that 35 days post-infection, 43 flies out of the 80 still alive were found to be *Tbg*2 PCR-positive in the saliva. These flies were able to infect 3 out of the 4 mice used for blood-feeding. Dissection revealed that only six flies in fact carried mature infections in their midguts and salivary glands. Importantly, a single fly with a mature infection was sufficient to infect a mammalian host. This *Tbg*2 transmission success confirms that *Tbg*2 strains can establish in tsetse flies and infect mammalian hosts. This study describes an effective *in vivo* protocol for transforming *Tbg*2 from procyclic to bloodstream form, reproducing the complete *Tbg*2 cycle from *G. p. gambiensis* to mice. These findings provide valuable insights into *Tbg*2’s host infectivity, and will facilitate further research on mechanisms of human serum resistance.

## Abstract (fr)

*Trypanosoma brucei gambiense* (*Tbg*) groupe 2 est un sous-groupe de trypanosomes capables d’infecter l’Homme, présent en Afrique de l’Ouest et en Afrique centrale. Contrairement aux autres agents responsables de la maladie du sommeil, tels que *Tbg* groupe 1 et *Trypanosoma brucei rhodesiense*, *Tbg*2 ne présente pas les marqueurs moléculaires habituellement associés à la résistance au sérum humain. Seules trente-six souches de *Tbg*2 ont été répertoriées, limitant considérablement les recherches sur ce sous-groupe malgré sa nature zoonotique. Certaines de ces souches ne sont disponibles que sous leur forme procyclique, ce qui freine la réalisation des tests de résistance au sérum humain et les études mécanistiques. De plus, la compréhension du potentiel de *Tbg*2 à infecter les glossines et les hôtes mammifères est limitée. Dans cette étude, 165 glossines *Glossina palpalis gambiensis* ont été infectées expérimentalement par des parasites *Tbg*2 sous leur forme procyclique. Trente-cinq jours après l’infection, 43 des 80 glossines encore en vie se sont révélées positives à *Tbg*2 en PCR sur leur salive. Ces glossines ont réussi à infecter trois des quatre souris utilisées pour leur repas de sang. La dissection des glossines a révélé que seules six d’entre elles étaient réellement porteuses d’infections matures dans leur intestin et leurs glandes salivaires. Il est important de noter qu’une seule glossine porteuse d’une infection mature a suffi pour infecter un hôte mammifère. Ce succès de transmission de *Tbg*2 confirme que les souches de *Tbg*2 peuvent s’établir dans les glossines et infecter des hôtes mammifères. Cette étude décrit un protocole *in vivo* pour transformer la forme procyclique de *Tbg*2 en forme sanguicole, en reproduisant le cycle complet de *Tbg*2 de *G. p. gambiensis* à la souris. Ces résultats fournissent des informations précieuses sur le potentiel infectieux de *Tbg*2 et faciliteront la recherche sur les mécanismes de résistance au sérum humain des souches.

## Introduction

*Trypanosoma brucei* (*Tb*) is an extracellular protozoan parasite transmitted by an arthropod hematophagous vector: the tsetse fly (*Glossina spp.*) [16]. Among the *Tb* species*,* the *Tb brucei* (*Tbb*) sub-species causes animal African trypanosomiasis or nagana in fauna. The human form of the disease, human African trypanosomiasis (HAT) or sleeping sickness is caused by the other two *Tb* sub-species: *Tb rhodesiense* (*Tbr*) and *Tb gambiense* (*Tbg*) [6]. *Tbr* causes an acute form of the disease in East Africa, whereas *Tbg* develops into a chronic form in Central and West Africa. *Tbg* HAT was responsible of 87% of the reported cases in 2019–2020 and is targeted by the World Health Organization for interruption of transmission by 2030 [9]. During the 1980s, the development of new analytical molecular methods allowed for the division of the *Tbg* subspecies into two groups. The most prevalent, genetically homogenous and monophyletic was group 1 (*Tbg*1) [11], invariably resistant to normal human serum (NHS) particularly thanks to the expression of the *Tbg-*specific glycoprotein (TgsGP) [2, 29]. In a recent review, group 2 (*Tbg*2) was defined as all human-infective *Tb* trypanosomes from West and Central Africa that do not fit into *Tbg*1 using various molecular markers [17]. *Tbg*2, *Tbb*, and *Tbr* are obviously highly diverse lineages but *Tbg*2 is different from *Tbr* with a consistent lack of serum resistance associated gene (SRA) [12]. If *Tbg*2 and its inconsistent resistance to lysis by human serum [21] does not appear to be a public health problem with only 36 strains referenced in the literature regarding the above definition, it represents a zoonotic form of HAT with a risk of transmission from animals to humans. In the current elimination context, it seems crucial to be able to detect such infections using adapted effective diagnosis and to determine if they are due to human serum resistance (HSR) trypanosomes or patient immunodeficiency (constitutive or transient) in order to implement adapted control strategies. *Tbg*2 stocks from different laboratories were gathered at UMR INTERTRYP (IRD/CIRAD, Montpellier, France) to study the HSR mechanisms and provide essential elements to anticipate the appearance of new mechanisms and to prevent possible phenomena of emergence [17].

*Tb* parasites have a multistage life cycle divided between the tsetse fly vector and a mammalian host. Along this life cycle, the parasite should continuously adapt to its surrounding environment. In the mammalian host, the bloodstream form (BSF) trypanosomes exist either in a proliferative long slender form or in a quiescent short stumpy form pre-adapted to the vector (Supplementary file 1). Following the infective blood meal, trypanosomes transform into their replicative procyclic form (PCF) in the tsetse fly midgut. Approximately one month after the infective meal, in a small proportion of tsetse flies (about 0.01% in natural conditions), trypanosomes colonize the salivary glands where they attach as epimastigote forms (EMF) [10]. Trypanosomes finally differentiate into infectious metacyclic forms (MCF) that can be transmitted to the mammalian host during the next blood meal.

Most of the *Tbg*2 strains are only available in their PCF and cannot be tested for their resistance to NHS, and for the study of the mechanisms implied. Moreover, very little is known about the potential for infection of *Tbg*2 in tsetse and mammalian hosts. Some rare studies have been conducted using PCF of *Tbg*1 or *Tbg*2, but transmissions to a mammalian host were not successful or were not attempted [25, 27]. The objectives of the present experimental study were (i) to confirm that *Tbg*2 PCF can settle in tsetse flies and become infectious to mice, and (ii) to transform *Tbg*2 PCF to BSF for further studies on HSR.

## Material and methods

### Ethics for animal experiments

Mice were kept under strict ethical conditions according to the international guidelines for the care and use of laboratory animals. The experiments designed for this study were approved by the Regional Ethics Committee for Animal Experimentation CEEA-LR 36 under project number APAFiS #34149 and authorized by the French Ministry for Higher Education and Research.

### Tsetse flies

In this study, tsetse flies were used from a colony of *G. p. gambiensis* originating from Burkina Faso and maintained at CIRAD (Montpellier, France). Only males were chosen, as they develop a higher proportion of salivary gland infections (mature infections) compared to females when experimentally infected with trypanosomes of the subgenus *Trypanozoon* [8, 19, 22]. Due to several issues relative to fly physiology (natural death, fluctuating infecting meal feedings, low rate of trypanosome colonization in the midgut and of mature infection in the salivary glands) already described elsewhere [20, 23], 165 tsetse flies were used assuming that this number would be sufficient to obtain mature infections after one month. Before the infection process, one wing was removed from each fly for security reason.

### Trypanosomes

**Figure 1:**
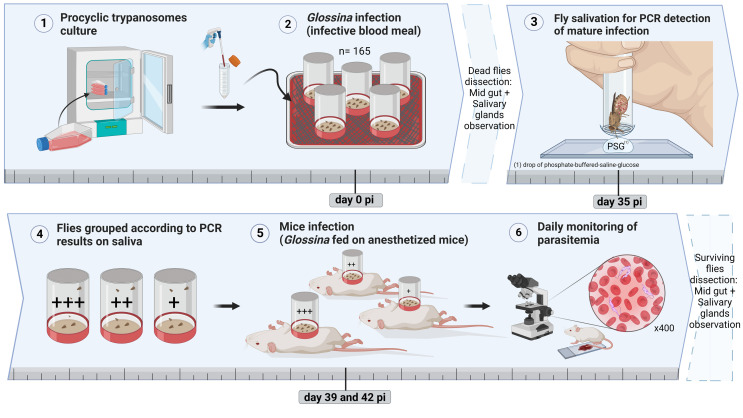
*Trypanosoma brucei gambiense* group 2 experimental *in vivo* cycle protocol (pi: post infection). +++: strong PCR signal; ++: medium PCR signal; +: weak PCR signal. PSG: phosphate-buffered saline-glucose.

The *Tbg*2 HTAG107-1 strain, also known as IPR107-1, was used in this study. This strain was isolated from humans in 1986 in the Daloa HAT focus, Côte d’Ivoire [28]. HTAG107-1 was grown in SDM-79 medium [3] supplemented with 20% fetal calf serum previously decomplemented (30 minutes at 56 °C). PCF trypanosomes were cultivated at 28 °C and harvested when 2.4 × 10^8^ parasites were obtained (1 × 10^7^ p/mL) (Figure 1). Parasites were then collected after centrifugation (1500×*g*, 10 minutes) and the pellet containing trypanosomes was washed three times with phosphate-buffered saline (PBS 1X) before use.

### Experimental infection of tsetse flies

PCF trypanosomes (2.4 × 10^8^) were gently mixed with 40 mL of sheep blood heated to 31 °C. The infected blood was proposed to starved *G. p. gambiensis* teneral males through a silicone membrane [1]. After the infective meal, tsetse flies were separated according to their blood-feeder status (blood meal visible in the abdomen or not). After 24 h, the process of infective meal was repeated with the non-gorged tsetse flies to ensure maximum infection rate success. Flies were then fed with uninfected sheep blood, three days a week for 35 days.

### Salivation assay and PCR

Thirty-five days after the infective meal (sufficient time to obtain mature infection in the salivary glands [25, 30]), surviving tsetse flies were individually subjected to a salivation test. Each fly was allowed to salivate into a drop of phosphate-buffered saline–glucose (PSG) on warmed slides (37 °C) [4, 14] and immediately placed into an individual cage. The salivate was recovered from the slide in 25 μL of sterile water (Figure 1). To identify flies whose saliva carried trypanosomes, DNA was extracted from each collected saliva and analyzed by TBR1/2 PCR, as already described [25]. Flies with PCR-positive saliva were then grouped in different cages according to PCR signal intensity (strong, medium, or weak) (Figure 1). Flies with PCR-negative saliva and flies whose salivate could not be recovered because flies refused to salivate were euthanized and dissected for microscopic observation (×400).

### Monitoring and dissection of the tsetse flies

Midguts of all flies found dead during the process were dissected from day 5 (time needed to observe parasite colonization of the midgut) to day 19 post-infection (pi). From day 20 pi, the salivary glands were also dissected (assuming that no trypanosomes can be found in the salivary glands before this time). All the dissected midguts and salivary glands were examined for trypanosomes by phase contrast microscopy at 400× magnification.

### Infection of mice

At day 39 and 42 pi, each group of flies with PCR-positive saliva was fed twice (3 days apart) on the belly of anesthetized female BALB/c mice previously immuno-suppressed with 0.15 mL of cyclophosphamide (ENDOXAN, 20 mg/mL) injected subcutaneously (Figure 1). A different mouse was assigned to each group of flies. The objective of this sorting was to maximize the success of infection of one of the mice. Tsetse were starved for three days prior to the mice blood meal to increase sting probability. The parasitemia of the mice was then determined daily by microscopy using the rapid “matching” method [15] on a drop of blood collected from the tail of each mouse (Figure 1).

The mice-fed surviving flies were euthanized and dissected for microscopic (×400) observation of the midgut and salivary glands.

## Results and discussion

### Molecular screening of tsetse flies with trypanosomes in their salivary glands

**Figure 2:**
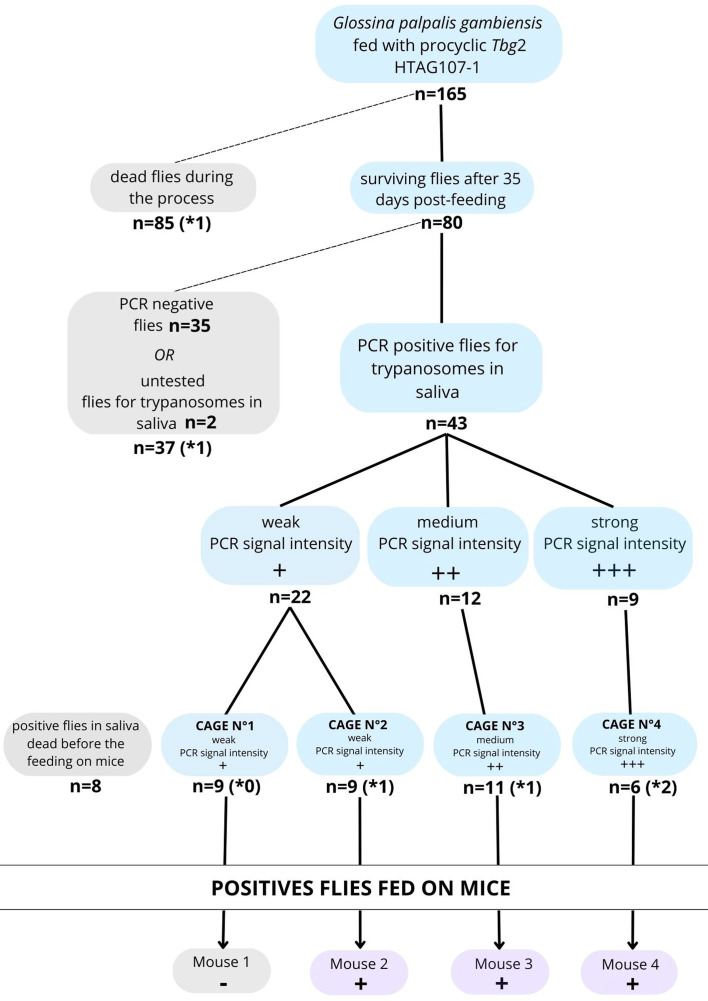
Diagram summarizing the main results of the experimental infection of flies and mice with *Trypanosoma brucei gambiense* group 2 HTAG-107 strain. (*x: number of flies found positive in salivary glands by microscopic observation after dissection).

Thirty-five days after tsetse fly infection, 80 flies out of 165 were still alive (Figure 2). Out of them, 78 were tested for the detection of trypanosomes in saliva by PCR and 43 (55%) showed PCR-positive saliva. Out of these 43 flies, 9, 12, and 22 exhibited a strong, medium, and weak PCR signal, respectively (Supplementary file 2). Between the beginning of the collection of the saliva and the results of the PCR analysis, 8 flies died. The remaining 35 flies were grouped into four cages: two containing 9 flies each with weak PCR signal, one containing 11 flies with medium PCR signal, and one containing 6 flies with strong PCR signal (Figure 2). All the PCR-negative flies were euthanized and dissected for microscopic observation of the midgut and salivary glands.

### Monitoring of tsetse fly infection

Throughout this experiment, 115 flies could be dissected of which 11 (9.6%) showed parasites in their midgut only, and six (5.2%) in both their midgut and salivary glands. Among the flies showing trypanosomes in their salivary glands that were fed on mice, none were found in cage No. 1, one was from cage No. 2, one from cage No. 3 and two from cage No. 4, in line with the molecular analysis.

The number of mature infections identified by the PCR analysis of the flies’ saliva (*n* = 43) was much higher than that determined by microscopic observation of the salivary glands (*n* = 6). These data are in the range found with previous observations from other studies (with maximum 10% flies found positive in SG) [25–27]. Because of its high sensitivity, PCR from saliva may offer a better view of mature infections as it is sometimes challenging to observe trypanosomes in salivary glands. However, for most of the PCR-positive flies for trypanosomes in their saliva, no trypanosomes were observed in the dissected salivary glands, despite diligent microscopic observation. Part of the PCR-positive saliva could result from flies only infected in the midgut, whose saliva may also contain regurgitated gut contents including trypanosome DNA.

Additionally, because the recovery of salivary glands is not easy during fly dissection, salivary glands may not always be available for observation. This was the case for three of the flies dissected at day 36 pi: trypanosomes were observed in the midgut only, but the salivary glands could not be recovered for technical reasons. Therefore, the percentage of flies with mature infection may have been underestimated through microscopy techniques.

### Monitoring infection in mice

Once the trypanosome PCR-positive flies from the four cages had been fed on mice, parasitemia was monitored daily. Three mice out of the four developed an infection two to four days after the blood meal (Figure 2 and Supplementary file 3). The resulting HTAG107-1 BSF trypanosomes were collected from the infected mice by cardiac puncture and supplemented with 50% glycerol before being stored in liquid nitrogen. Dissection and observation of the salivary glands of the flies used to bite the mice showed that two flies were positive for trypanosomes in cage No. 4, one in cage No. 3, and one in cage No. 2. No salivary gland-positive flies were detected in cage No. 1, which is congruent with the absence of infection in mouse 1.

### Advantages and drawbacks to reconstitute a PCF to BSF experimental *in vivo* life cycle

From 165 flies fed with a single meal of sheep blood mixed with cultivated PCF trypanosomes, at least 6 flies with mature infections after one month were obtained.

We succeeded in infecting mice from infected tsetse flies. This achievement is partly due to the large size of the starting sample (*n* = 165) and to the starvation of the flies before the infective meal. Post-dissection of the flies used to infect the mice showed that a mouse only needs to be bitten by one fly with mature infection to become infected. This success also confirms that *Tbg*2 group strains can settle in the tsetse fly and infect a mammalian host.

For strains only available as PCF, the results of this experiment made it possible to obtain the bloodstream form that can be evaluated for resistance to NHS. However, this experiment is time consuming, requires great technical effort and is not in line with current animal ethics principles (3R rule – replace, reduce, refine). For this reason, if the passage from PCF to BSF is the only result desired, *in vitro* plasmid methods should be preferred [24].

While HAT elimination seems a realistic goal for 2030, we advocate for improving knowledge of the *Tbg* strains that are still circulating, even at a low, almost undetectable level. In several HAT foci, parasitemia observed in human or in animal reservoirs is very low [7] and hinders deep genotypical and phenotypical characterization. Isolating strains and mastering a transmission cycle makes it possible to collect data that are not available otherwise, for instance to account for differences in pathogenicity and virulence to humans over natural cycles [5]. This is particularly interesting in the case of *Tbg*2 strains. They have been found to be resistant or partially resistant to the NHS, but their ability to maintain NHS resistance capacity after cycling in animals is unknown. Deciphering the nature of the resistance to NHS – constitutive (as is the case for *Tbg*1) or conditional (which can be lost after several vector/animal cycles) – is keystone information for the effective and sustainable elimination of sleeping sickness. Indeed, elimination of HAT will lead to a decrease in acquired immunity in populations, which could create major concerns for more susceptible populations if they are again exposed to strains with constitutive resistance [17].

Finally, it remains difficult to reproduce complete cyclical transmission (from infection of the tsetse fly to transmission to the host by the tsetse fly) of *Tbg*2 and even *Tbg*1 because of the low rate of mature infections of *Tbg* [20]. Two other studies succeeded using clones of *Tbg*2 BSF and *Glossina morsitans* [13, 18]. In this study, we provide evidence of an effective *in vivo* protocol to transform *Tbg*2 PCF to BSF by experimentally reproducing the complete *Tbg*2 cycle from *G. p. gambiensis* to the mouse.

## Figures and Tables

**Supplementary file 1:**
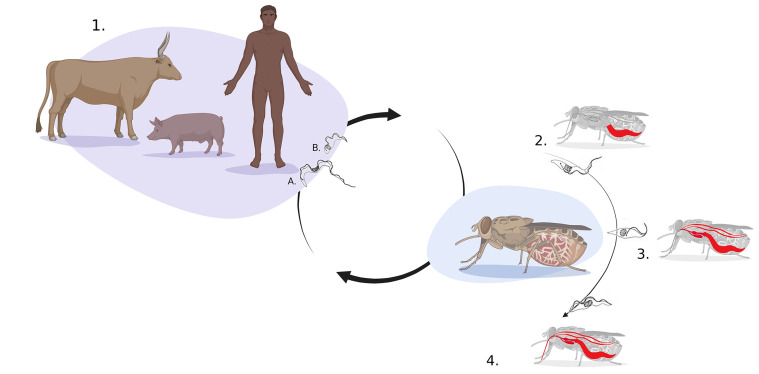
Schematic life cycle of *Trypanosoma brucei*. 1: Bloodstream trypanosomes in mammalian host; A: long slender form; B: short stumpy form. 2: Procyclic trypanosomes in tsetse fly midgut. 3: Epimastigote trypanosomes colonize salivary glands. 4: Metacyclic trypanosomes in salivary glands can be transmitted to a mammalian host during the next tsetse fly meal. In red: localization of the parasites in the tsetse fly through the cycle.

**Supplementary file 2:**
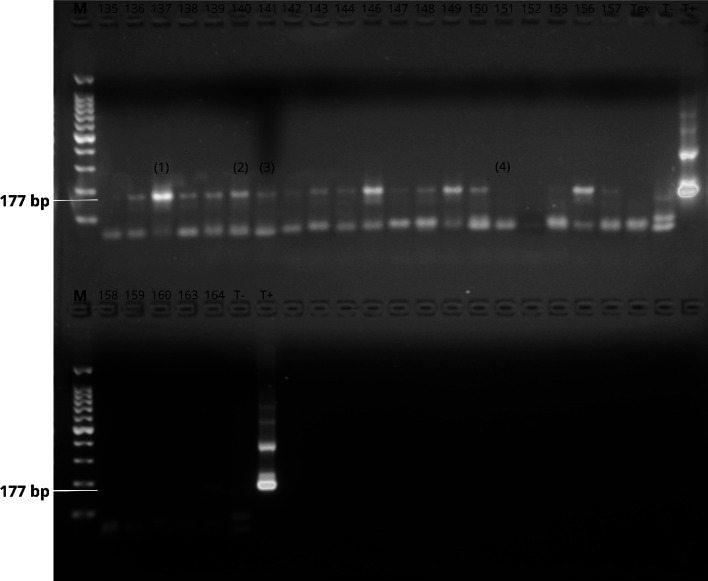
TBR1/2 PCR identification of *Trypanosoma brucei s.l* in tsetse fly saliva showing 177 bp DNA satellite repeat specific for *T. brucei s.l* – numbers correspond to those assigned to the flies. M: 100 bp DNA size marker; Tex: DNA extraction negative control; T−: PCR-negative control; T+: positive control; (1): Strong PCR signal; (2): Medium PCR signal; (3): Weak PCR signal; (4): Negative PCR signal.

## Supplementary material

The Supplementary materials of this article are available at https://www.parasite-journal.org/10.1051/parasite/2024009/olm.

*Supplementary file 3*: Microscopic observation videos of *Trypanosoma brucei gambiense* group 2 throughout its life cycle, including (1) procyclic form in tsetse midgut, (2) metacyclic form in tsetse salivary glands, and (3) bloodstream form found in infected mice blood.
